# Plasticity in the developing brain: neurophysiological basis for lesion-induced motor reorganization

**DOI:** 10.1093/braincomms/fcab300

**Published:** 2021-12-21

**Authors:** Mitchell Batschelett, Savannah Gibbs, Christen M. Holder, Billy Holcombe, James W. Wheless, Shalini Narayana

**Affiliations:** 1 Neuroscience Institute, Le Bonheur Children’s Hospital, Memphis, TN, USA; 2 Rhodes College, Memphis, TN, USA; 3 Department of Pediatrics, Division of Pediatric Neurology, College of Medicine, University of Tennessee Health Science Center, Memphis, TN, USA; 4 Department of Anatomy and Neurobiology, College of Medicine, University of Tennessee Health Science Center, Memphis, TN, USA

**Keywords:** motor mapping, transcranial magnetic stimulation, reorganization, cortical dysplasia, peri-natal brain injury

## Abstract

The plasticity of the developing brain can be observed following injury to the motor cortex and/or corticospinal tracts, the most commonly injured brain area in the pre- or peri-natal period. Factors such as the timing of injury, lesion size and lesion location may affect a single hemisphere’s ability to acquire bilateral motor representation. Bilateral motor representation of single hemisphere origin is most likely to occur if brain injury occurs before the age of 2 years; however, the link between injury aetiology, reorganization type and functional outcome is largely understudied. We performed a retrospective review to examine reorganized cortical motor maps identified through transcranial magnetic stimulation in a cohort of 52 patients. Subsequent clinical, anthropometric and demographic information was recorded for each patient. Each patient’s primary hand motor cortex centre of gravity, along with the Euclidian distance between reorganized and normally located motor cortices, was also calculated. The patients were classified into broad groups including reorganization type (inter- and intrahemispheric motor reorganization), age at the time of injury (before 2 years and after 2 years) and injury aetiology (developmental disorders and acquired injuries). All measures were analysed to find commonalities between motor reorganization type and injury aetiology, function and centre of gravity distance. There was a significant effect of injury aetiology on type of motor reorganization (*P* < 0.01), with 60.7% of patients with acquired injuries and 15.8% of patients with developmental disorders demonstrating interhemispheric motor reorganization. Within the interhemispheric motor reorganization group, ipsilaterally and contralaterally projecting hand motor cortex centres of gravity overlapped, indicating shared cortical motor representation. Furthermore, the data suggest significantly higher prevalence of bilateral motor representation from a single hemisphere in cases of acquired injuries compared to those of developmental origin. Functional outcome was found to be negatively affected by acquired injuries and interhemispheric motor reorganization relative to their respective counterparts with developmental lesions and intrahemispheric motor reorganization. These results provide novel information regarding motor reorganization in the developing brain via an unprecedented cohort sample size and transcranial magnetic stimulation. Transcranial magnetic stimulation is uniquely suited for use in understanding the principles of motor reorganization, thereby aiding in the development of more efficacious therapeutic techniques to improve functional recovery following motor cortex injury.

## Teaser

Batschelett *et al*. describe corticomotor reorganization findings in a paediatric cohort using transcranial magnetic stimulation. The authors found acquired brain injuries (e.g. stroke) are more likely than developmental disorders (e.g. polymicrogyria) to induce interhemispheric reorganization and produce poor motor function. Additionally, corticomotor representation appears to be shared following interhemispheric reorganization.

## Graphical Abstract

**Graphical Abstract:**
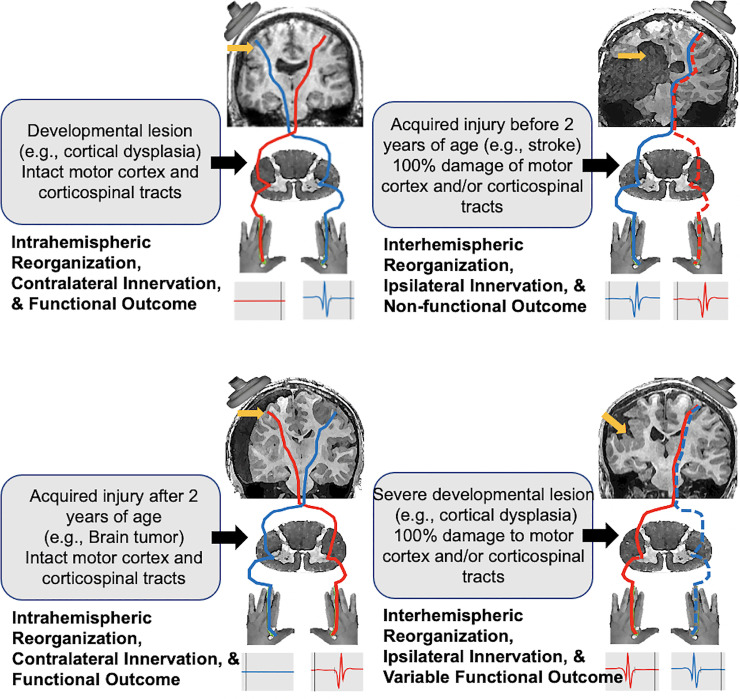

## Introduction

The human brain possesses the intrinsic ability to reorganize and recover function following injury and/or developmental malformations. This reorganizational capability, or plasticity, is observable throughout the human lifespan. For example, many adult stroke patients exhibit post-injury motor cortex plasticity and partial recovery of motor function.^1–3^ However, the developing brain displays a greater capacity to recover following injury compared to its adult counterpart.^4–7^ Cortical plasticity in the developing brain is readily observed in the case of motor cortex injury, as the motor cortex and/or corticospinal tract is a common site of brain damage, particularly in the pre- or immediately peri-natal period. Therefore, studying motor reorganization in children following injury to the motor cortex and/or corticospinal tract provides an excellent surrogate to understanding basic mechanistic principles of cortical reorganization.

Although the increased plasticity of the developing brain is well documented,^8,9^ the exact mechanistic principles that underlie cortical motor reorganization are largely understudied. In humans, it has been shown that the developing brain includes fast and direct ipsilateral corticospinal projections from both hemispheres until ∼24 months of postnatal development,^10–14^ which disappear following full, unaltered corticospinal maturation. Longitudinal and cross-sectional neurophysiological studies of typically developing infants and children are likewise consistent with the withdrawal of uncrossed corticospinal axons over the first 24 postnatal months, such that ipsilateral motor evoked potentials (MEPs), like those elicited by transcranial magnetic stimulation (TMS), are less frequent, smaller, later in onset and have higher thresholds compared to contralateral muscle responses at 2 years of age.^11,15^

Injury to the motor cortex in young children alters the normal development of the motor cortex and/or corticospinal tract. Generally, two mechanisms of cortical reorganization have been postulated: (i) interhemispheric reorganization (IEHR), where the function is transferred to contralateral homologues and (ii) intrahemispheric reorganization (IAHR), where function is taken over by residual tissue and nearby cortex in the lesioned hemisphere.^10,16^ It is thought that in the case of IEHR, the direct ipsilateral corticospinal projections of the unaffected hemisphere fail to regress, but rather persist, subsequently shifting the lesioned hemisphere’s motor control to homologous ipsilateral motor areas.^12,13,17,18^ The exact factors that govern which type of reorganization occurs are largely unknown; however, it is documented that lesion timing, size and location play a significant determining role.^5,7,16,19–23^ For example, numerous studies have found that IEHR prevalence decreases with age, indicating that lesions early in life promote IEHR, whilst IAHR is primarily observed in later occurring lesions.^12,19,21,24,25^ This age-dependent reorganizational effect suggests that the maturational status of the motor system at the time of injury dictates, in part, the resulting pattern of reorganization.

Other studies have observed a link between lesion size and subsequent reorganization. In short, larger perirolandic lesions are more likely to evoke IEHR, whilst smaller lesions, despite being around the rolandic sulcus, are more likely to evoke IAHR.^12,13^ Additionally, other studies have found that lesion size is not a complete predictor of reorganization type, as large but incomplete rolandic lesions showed an increased incidence of IAHR than complete rolandic lesions of similar size.^20^ These results demonstrate that IEHR is more likely to occur if the lesion affects the totality of the motor cortex early in life, forcing function to shift to the motor homologues of the contralesional hemisphere.

The severity of damage to descending white matter tracts early in development has also been implicated as a potential driving factor in determining reorganization. Namely, white matter damage has been shown to be positively correlated with the incidence of IEHR whilst negatively affecting motor function.^19^ Consistent with these data, it was also found that greater injury to descending white matter tracts coincided with increased ipsilateral motor cortex recruitment and degree of motor reorganization.^26^ Reports in regard to motor function are more conflicted, as the levels of functional impairment due to IEHR and IAHR have often been indistinguishable.^17,27^

Although the link between reorganization type, lesion timing and size has been documented, the role of lesion aetiology on induced reorganization type is largely understudied, as only a few case reports or smaller sample sizes (*N* ≤ 10) have examined this aspect.^27–29^ Further, though studies indicate a shift to contralateral homologues,^6,13^ the precise location of reorganized motor cortex in IEHR has never been directly measured. The present study retrospectively examined motor reorganization as indexed by TMS in a cohort of patients presenting with acquired injuries or developmental disorders. We examined the effect of lesion aetiology (acquired versus developmental) on the type of reorganization (IEHR versus IAHR). Moreover, we measured the Euclidean distance between the centre of gravity (COG) of normally located and reorganized motor cortices within subjects to further characterize the extent of reorganization and determine the overlap of motor cortices following IEHR. We also examined the relationship between white matter damage and reorganization, as well as the resulting functional implications of reorganization type, as there are conflicting reports on which type of reorganization yields the best functional outcome.^5,19,23,30^ In addition to providing novel information regarding the mechanisms of corticomotor reorganization, this study serves as evidence for the efficacy of TMS in examining motor reorganization in a paediatric cohort.^31,32^

We hypothesized that acquired injuries would be more likely to induce IEHR than developmental disorders and that cortical real estate would be shared in the case of IEHR. We also expected that increased white matter damage would drive IEHR. Finally, we predicted that the best functional outcome would occur following IAHR observed in developmental disorders.

## Materials and methods

### Study cohort

**Figure 1:**
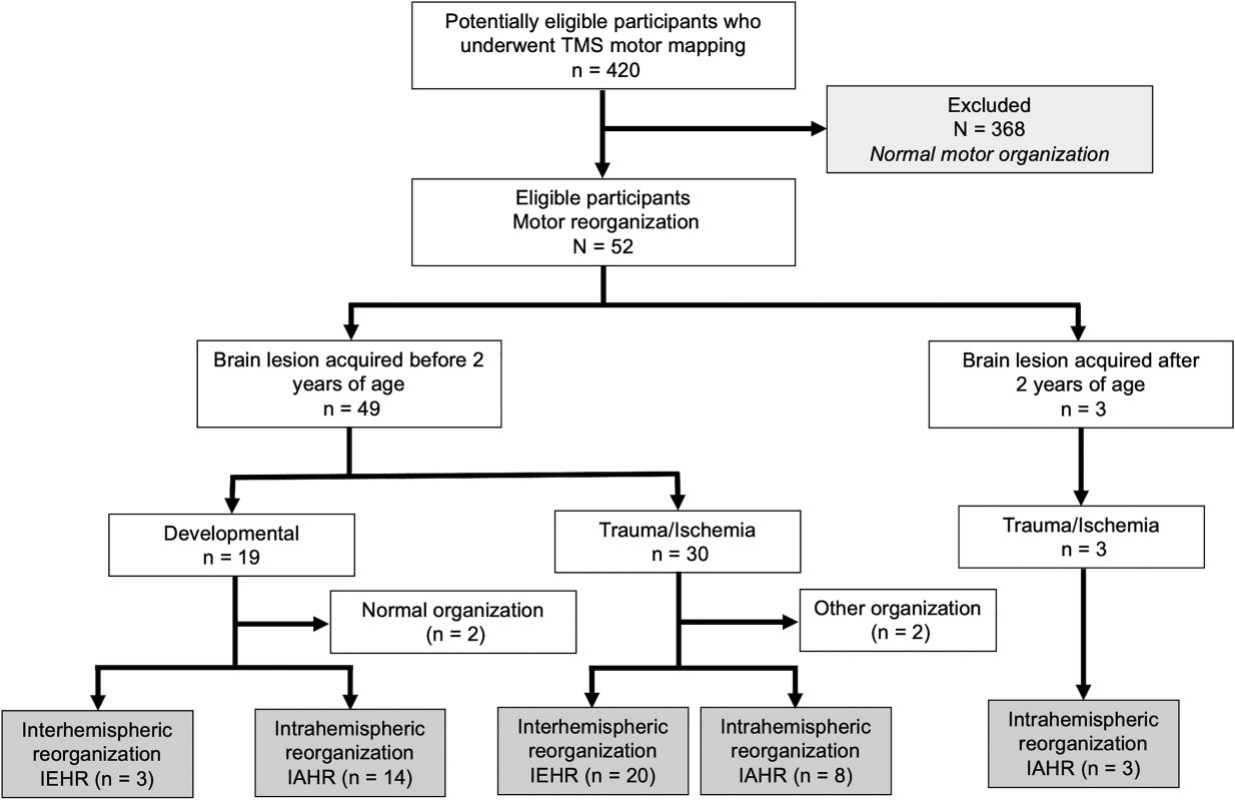
**Flow chart for identifying the study cohort.** IEHR, interhemispheric reorganization; IAHR, intrahemispheric reorganization.

Through a retrospective chart review, we identified 420 patients who underwent TMS motor mapping at Le Bonheur Children’s Hospital between July 2012 and May 2019. This study was approved by the institutional regulatory boards of the University of Tennessee Health Science Center and Le Bonheur Children’s Hospital. Fifty-two patients were deemed eligible for inclusion in this study (Fig. 1 and Table 1). Eligible patients were those in whom either the cortical motor map of the primary hand motor cortex was displaced from the central sulcus, indicating IAHR, or at least one hemisphere demonstrated abnormal ipsilateral corticospinal projections after the accepted period of ipsilateral projection regression, indicating IEHR. More specifically, IAHR determinations were made based upon previous TMS investigations delineating normal hand motor cortex location and distribution.^33–39^ With these studies in mind, IAHR was deemed present if at least one hemisphere exhibited motor cortex representation either within cortical regions known to be associated with the non-hand motor function (i.e. leg or face motor cortex)^39^ or anteriorly or posteriorly displaced from the precentral gyrus, outside of the known normal deviation of hand motor cortex area (i.e. anterior to the middle frontal gyrus or posterior to the postcentral gyrus).^33–38^ Patients who demonstrated normal motor maps despite lesions in the motor pathway, typically either small or in the subcortical regions, were not included in this study. Eligibility determinations were made using visualized cortical motor maps of the primary hand motor cortices following presurgical and/or clinically indicated TMS motor mapping, as well as a retrospective chart review, including demographics, clinical history, previous brain imaging, neuropsychological testing and, in some cases, physical and occupational therapy evaluations. For each patient included in the study, the following data were recorded: sex, date of birth, age at the time of motor mapping, type of brain lesion, lesion location, lesion size relative to motor cortex (<50% involvement, >50% but incomplete involvement or complete motor cortex damage), age incurred (before or after 2 years of age), motor cortex reorganization type (IEHR or IAHR), location of resulting reorganization (only recorded for IAHR patients), history of epilepsy and grasp function (non-functional or functional). Antiepileptic medications (AEDs) prescribed at the time of TMS data acquisition were also collected. Based on brain injury aetiology, subjects were placed into two broad groups: developmental brain disorders (i.e. cortical dysplasia, heterotopia, polymicrogyria, etc.) and acquired injuries (i.e. traumatic brain injury, stroke, tumour resection, hemispherectomy, lobectomy, etc.). Groupings were designed to amplify potential links between groups, including type and extent of reorganization and functional outcome patterns. The aforementioned variables and their relationships were further examined via statistical testing (see the Statistical analysis section).

**Table 1:** Study cohort demographics

	Injury aetiology: developmental	Injury aetiology: acquired injury	Total
Number of patients	19	33	52
Age at the time of testing (years, mean ± SD)	9.7 ± 5.1	11.0 ± 8.8	10.5 ± 7.6
Age range (years)	1.7–19.1	1.7–50	1.7–50
Gender: females/males	10/9	21/12	31/21
Lesion acquisition: before age 2/after age 2	19/0	30/3	49/3
Lesioned hemisphere: right/left/bilateral	12/4/3	15/16/2	27/20/5
Interhemispheric motor reorganization^a^	3	*20* *	23
Intrahemispheric motor reorganization^a^	*14* *	11	25
No demonstrable reorganization	*2*	2	4

Italics indicate significant difference between the two groups. SD, standard deviation.

^a^ Injury aetiology was found to have a significant effect on the resulting type of corticomotor reorganization, with developmental disorders mainly result in intrahemispheric motor reorganization, whilst acquired brain injury primarily results in an interhemispheric motor reorganization.

* *P* < 0.01.

### Transcranial magnetic stimulation

#### TMS motor mapping procedure

**Figure 2:**
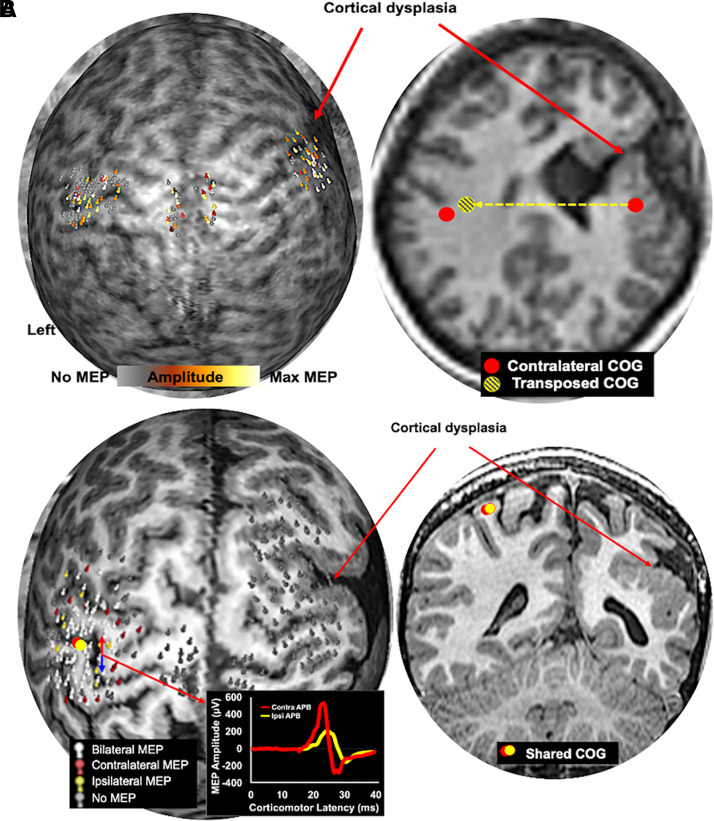
**Examples of motor reorganization in developmental cohort**. (**A**) A 19-year-old female with focal right hemisphere polymicrogyria demonstrating an IAHR pattern. The right hemisphere motor cortex is localized directly over the area of polymicrogyria and was displaced anteromedially when compared with the contralateral motor cortex. (**B**) An 11-year-old male with extensive right hemisphere polymicrogyria demonstrating a rare case of IEHR pattern. The descending white matter tract in the right hemisphere was also affected, making them non-functional. Hence, a shared bilateral corticomotor representation was observed in the left hemisphere. An example of bilateral MEP elicited by stimulation the motor cortex in the left hemisphere is shown.

All individuals underwent TMS motor mapping either as part of their Phase I evaluation for refractory epilepsy, functional mapping prior to brain tumour surgery or to elucidate the functional state of their motor cortex following injury. Motor mapping was performed using a 70 mm figure-of-eight coil integrated into the navigated TMS system (Nexstim Plc., Helsinki, Finland) having a maximum electrical field of 172 V/m at 25 mm from its surface. The high-resolution T1-weighted MRI, the patient and the TMS coil were coregistered using a 3D tracking system. The MEP elicited by TMS was recorded by surface EMG from bilateral abductor pollicis brevis (APB) muscles using disposable electrodes (Neuroline 720, Ambu Inc., MD, USA) and sampled at 3 kHz and band-pass filtered from 10 to 500 Hz. In individuals who could maintain a quiet EMG baseline (*N* = 37), the resting motor threshold (rMT) for both hemispheres were measured at the hotspot for APB using an automated algorithm implemented in the Nexstim software based on the guidelines of the International Federation of Clinical Neurophysiology^40^ and expressed as percent maximum stimulator output (%MSO). The extent of the motor cortex was then mapped at a TMS intensity of 110% of rMT; brain areas where an MEP ≥50 µV amplitude was elicited were included in the map and shown as a heat map (example shown in Fig. 2A). In patients who could not maintain a quiet EMG baseline or experienced pain during stimulation (*N* = 7), the MEP amplitudes were visually assessed and the motor cortex was mapped using the TMS intensity that elicited MEP amplitudes ≥50 µV. These patients were not included in the rMT analysis. All patients tolerated TMS without any serious adverse effects. Each patient’s TMS session, containing each stimulation location, intensity and resulting MEP, was reviewed. A stimulation was considered to be a valid representation of the hand motor cortex and its corticospinal projection if it elicited a triphasic/polyphasic MEP with an amplitude ≥50 µV. Additionally, corticomotor latencies were measured as time from TMS stimulation to MEP onset for each hemisphere in the IAHR group and for both extremities from the intact hemisphere within the IEHR group. Five patients from the IEHR and eight patients from the IAHR group were excluded from the analysis due to insufficient corticomotor latency data.

#### COG of motor maps

Rather than selecting the site where the MEP amplitude was highest, cortical representation for APB was defined by the COG. The COG is largely agnostic to MEP amplitude variabilities and has been shown to be a more accurate representation of the motor cortex.^41^ The cortical location where an MEP was elicited in the APB and the MEP amplitude was used to calculate the COGs using the formula:

(1) $$\sum a_{i}x_{i}/\sum a_{i};\;\sum a_{i}y_{i}/\sum a_{i};\;\sum a_{i}z_{i}/\sum a_{i}$$

where *x*_*i*_ is the mediolateral location; *y*_*i*_ theanteroposterior location; *z*_*i*_ the superoinferior position and *a*_*i*_ the MEP amplitude at that location.^42^ A measure of the distance between the normally located and reorganized COGs was necessary to observe the nature of cortical reorganization in the lesioned hemisphere relative to the normally located hand motor area within subjects. The primary hand motor cortex in the contralesional hemisphere acted as a within-subject control. For those in the IEHR group, the COGs of the hemisphere’s ipsilateral projections and contralateral projections, both localized within the intact hemisphere, were calculated independently. For those in the IAHR group, the COG for APB representation was calculated independently for the two hemispheres; then, the COG in the lesioned hemisphere was transposed onto the intact hemisphere by mirroring its location around the midline. With the two COGs localized to the same hemisphere, the absolute distance between the COGs of the reorganized motor cortex and the normally located motor cortex was calculated by finding the Euclidian distance (mm) between them using the formula:

(2) $$d=\sqrt{(x_{2}}-x_{1}{)}^{2}+(y_{2}-y_{1}{)}^{2}+(z_{2}-z_{1}{)}^{2}$$

The COG distance values were averaged for each group and the difference between the means was examined to assess the nature of IEHR and IAHR patterns. COG data from five patients were unavailable due to insufficient EMG data.

### Hand function

The hand function status in each patient was derived from a review of the hand motor assessment subsection of the neuropsychological evaluation. The most commonly used tests to examine the functionality of the affected hand were the Purdue Pegboard, Grooved Pegboard, Bayley Scales of Infant and Toddler Development (3rd edition) and Mullen Scales of Early Learning. The relationship between the time of injury, size of injury, location of injury, aetiology of injury and residual hand function were assessed. Overall, two patients had insufficient data to make an assessment about grasp function.

### White matter tract analysis

The integrity of the white matter tracts in the study cohort was evaluated by visually examining the subjects’ high-resolution T1-weighted anatomical MRIs. The hand motor cortex COG coordinates were marked on the MRI. Descending white matter tract viability from the COGs was examined qualitatively. Furthermore, the cerebral peduncle, a region containing descending corticospinal motor neurons,^43^ was examined for possible asymmetry.

### Lesion size

The size of each patient’s lesion was assessed and documented relative to the amount of lesion involvement within the accepted area of hand motor representation.^33–38^ Lesions were qualitatively characterized as <50% involvement, >50% but incomplete involvement and complete motor cortex involvement. Possible effects of lesion size on resulting reorganization and grasp function were examined (see the Statistical analysis section).

### Statistical analysis

All statistical analyses were conducted using SPSS (Release 27.0.1.0; IBM Corporation, Armonk, NY, USA). The effect of injury aetiology on reorganization type was assessed using Fisher’s exact test. One-tailed paired *t*-tests were conducted for rMT differences between lesioned and non-lesioned hemispheres for the IAHR group and between contralateral and ipsilateral projections within the same hemisphere for the IEHR group. Additionally, two-tailed paired *t*-tests were conducted to examine differences in corticomotor latency in both groups. The distance between the COGs of reorganized and normally located APB in the IEHR and IAHR groups was examined using a two-tailed, two-sample *t*-test for unequal variance. Fisher’s exact test was conducted to test for an effect of reorganization type on motor function and the effect of lesion aetiology on motor function. Finally, *χ*^2^ testing was also conducted to examine possible effects of lesion size on resulting reorganization and grasp function.

### Data availability

The data that support the findings of this study are available from the corresponding author upon request.

## Results

### Study cohort

**Figure 3:**
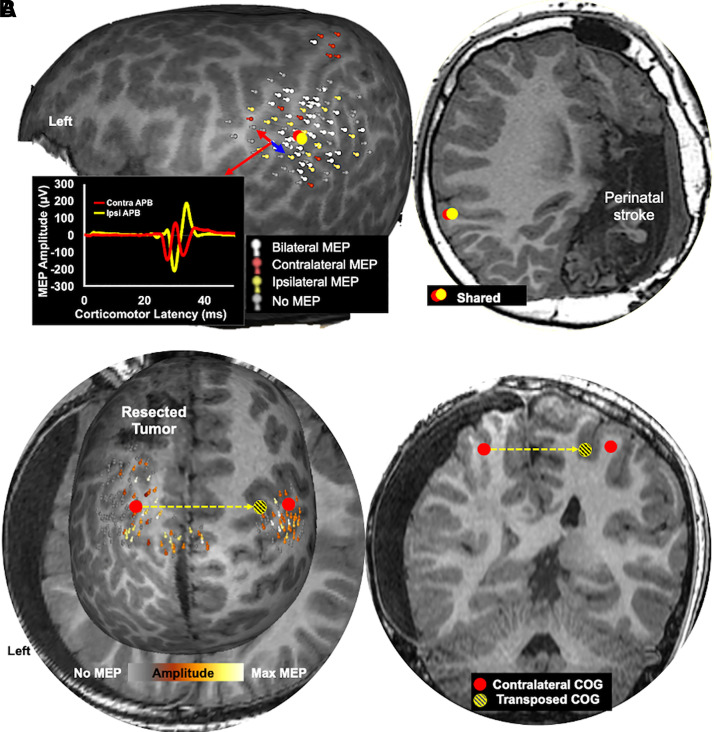
**Examples of motor reorganization in acquired brain injury cohort.** (**A**) A 16-year-old male with a history of intraparenchymal haemorrhage at birth and secondary epilepsy. The brain insult caused complete damage to right hemisphere motor cortex including its white matter tracts. No motor representation was observed in this hemisphere and a shared bilateral corticomotor representation, i.e. IEHR was observed in the left hemisphere. An example of bilateral MEP elicited by stimulation the motor cortex in the left hemisphere is shown. The patient also had severe global developmental delays, including both motor and cognitive deficits. (**B**) An 11-year-old male with a history of left hemisphere frontal lobe tumour located anterior to the primary motor cortex. His seizures began before the age of 2 years, secondary to the tumour. The primary hand motor cortex in the left hemisphere demonstrated an IAHR pattern and was displaced medially when compared with the homologue in the right hemisphere.

The demographic, clinical and motor reorganization patterns observed in the study cohort are tabulated in Table 1 (see Supplementary Tables 1 and 2 for more detailed clinical information). The cohort consisted of 31 females and 21 males with an average age of 10.5 ± 7.6 years. Of the 52 patients in this study, 49 had sustained brain lesion at birth or before 2 years of age (19 presented with developmental disorders and 30 suffered an acquired injury; Fig. 1 and Table 1). The remaining three patients acquired the brain lesion when they were older than 2 years. Twenty-seven individuals had lesions in the right hemisphere, 20 in the left hemisphere and five had bilateral or non-focal lesions. Twenty-five patients demonstrated IAHR and 23 demonstrated IEHR (see Figs 2 and 3 for examples). Four patients (two with developmental and two with acquired lesions) with no clear reorganization were included in the cohort, as they presented with abnormally non-localizable motor cortices. These patients were only factored into analyses relating to effects of lesion aetiology and lesion size on functional outcome. Of those demonstrating IAHR, the majority (56%) had a developmental lesion (Table 1 and Fig. 2A), whilst the majority of patients (87%) in the IEHR group had an acquired injury (Table 1 and Fig. 3A). See Tables 1–3 for complete cohort description. Of the three patients with brain injury occurring after age 2 years, all were within the acquired group, and all demonstrated IAHR (Supplementary Table 1).

**Table 2:** Grasp function and TMS parameters in the two injury aetiology groups

	Injury aetiology: developmental	Injury aetiology: acquired injury
Number of patients	19	33
Grasp function: non-functional	8 (42%)	24 (73%)
Grasp function: functional^a^	10 (53%)	8* (24%)
Grasp function: insufficient information	1 (5%)	1 (3%)
TMS intensity: lesioned hemisphere (% MSO)^b^	87.1 ± 17.3*	72.4 ± 26.1
TMS intensity: non-lesioned Hemisphere (% MSO)^b^	75.2 ± 23.5	66.3 ± 25.8

MSO, maximum stimulator output.

^a^ The developmental brain injury was significantly more likely to produce functional grasp when compared with acquired brain injury.

^b^ TMS intensity to elicit a motor response was significantly higher in the lesioned hemisphere in developmental brain injury aetiology.

* *P* < 0.05.

**Table 3:** Grasp function, TMS and COG parameters in the two patterns of motor reorganization

	Interhemispheric reorganization	Intrahemispheric reorganization
Number of patients	23	25
Gender: females/males	15/8	12/13
Grasp function: non-functional	19 (83%)	10 (40%)
Grasp function: functional^a^	2 (9%)	15** (60%)
Grasp function: insufficient information	2 (9%)	0 (0%)
TMS intensity: lesioned hemisphere (% MSO)^b^	n/a	81.0 ± 22.2*
TMS intensity: non-lesioned Hemisphere (% MSO)^b^	n/a	71.5 ± 24.4
TMS intensity: contralateral projections (% MSO)^c^	71.3 ± 24.6	n/a
TMS intensity: ipsilateral projections (% MSO)^c^	77.6 ± 21.6*	n/a
COG Euclidian distance APB (mm)^d^	2.7 ± 1.7**	16.0 ± 8.6

COG, centre of gravity; MSO, maximum stimulator output.

^a^ An intrahemispheric reorganization was significantly more likely to produce functional grasp function.

^b^ TMS intensity required to elicit a motor response was significantly higher in the lesioned hemisphere for individuals in the IAHR group.

^c^ TMS intensity required to elicit a motor response was significantly higher for ipsilateral projections than for contralateral projections within the non-lesioned hemisphere demonstrating interhemispheric reorganization.

^d^ The centres of gravity of the normally located and reorganized representation for APB were significantly closer for persons in the IEHR group than for individuals in the IAHR group.

* *P* < 0.05.

** *P* < 0.0001.

Of the whole study cohort, 44 patients (85%) were prescribed at least one AED. Of those taking AEDs, most (40%) were prescribed two. The most common AEDs were oxcarbazepine and levetiracetam. There were no across-group differences in either the number of patients on AEDs or the number of AEDs prescribed (see supplemental Tables I and II for details).

*χ*^2^ testing conducted to measure an effect of lesion size on resulting reorganization did not achieve significance (*P* = 0.611); however, only incomplete lesions (i.e. lesions with motor cortex involvement of <50% or >50% but incomplete) were included in this analysis, as complete motor cortex lesions (*N* = 8) always resulted in IEHR. Taken together, these results suggest that complete motor cortex lesions significantly contribute to IEHR, whilst any type of incomplete lesion does not significantly contribute an effect on resulting reorganization.

Fisher’s exact test conducted to measure injury aetiology’s effect on reorganization type, independent of injury timing and overall age, found a significant effect of injury aetiology on resulting corticomotor reorganization. Acquired injuries were significantly more likely to cause IEHR than developmental disorders (*P* < 0.01) whereas occurance of IAHR was signifcantly higher in developmental disorders (*P *< 0.01). Fisher’s exact test conducted to measure gender effects on reorganization type was not significant (*P* = 0.38), demonstrating that gender does not play a role in induced corticomotor reorganization.

### Transcranial magnetic stimulation

#### Motor threshold

The rMT data were available in 37 patients. rMT in the lesioned hemisphere (87.1 ± 17.3%) was higher than rMT in the non-lesioned hemisphere (75.2 ± 23.5%) in the developmental, but not the acquired injury cohort (Table 2). The average rMTs in the acquired injury group were 72.4 ± 26.1% and 66.3 ± 25.8% for lesioned and non-lesioned hemispheres, respectively. In the IAHR group, the average rMT in the lesioned hemisphere was 81.0 ± 22.17% MSO, compared to 71.5 ± 24.4% MSO in the intact hemisphere (Table 3). In the IEHR group, average rMT for ipsilateral projections was 77.6 ± 21.6% MSO, whilst average rMT for contralateral projections was 71.3 ± 24.6% MSO (Table 3). One-tailed paired *t*-tests conducted for rMT differences between lesioned and non-lesioned hemispheres within the IAHR group found lesioned hemispheres to have significantly higher rMTs than non-lesioned hemispheres (*P* = 0.01). One-tailed paired *t*-tests conducted for rMT differences between contralateral and ipsilateral projections within subjects and within hemispheres for the IEHR group found ipsilateral projections to have significantly higher rMTs than contralateral projections (*P* = 0.01; Table 3).

#### Corticomotor latencies

In the IEHR group (*n* *=* 22), average corticomotor latencies were 21.45 ± 2.12 and 21.02 ± 2.11 ms for ipsilateral and contralateral projections, respectively. In the IAHR group (*N* = 19), corticomotor latencies were 22.16 ± 4.25 and 20.96 ± 2.60 ms for affected and unaffected hemispheres, respectively. The difference of the mean two-tailed *t*-tests did not reveal differences in corticomotor latency between ipsilateral and contralateral projections or affected and unaffected hemispheres for the IEHR and IAHR groups, respectively.

#### COG of motor maps

The average distance between the COGs for APB muscles in the two hemispheres was 16.0 ± 8.6 mm in IAHR (Table 3). For the IEHR group, the average distance between the COGs for APB muscles was 2.7 ± 1.7 mm. See Tables 2 and 3 for a complete listing of COG and TMS parameters. A two-tailed, two-sample of unequal variance *t*-test examining the average distances between reorganized and normally located APB COGs between the IEHR and IAHR cohorts found that the distances were significantly shorter for the IEHR group than the IAHR group (*P* = 5.08E−08), indicating a significant overlap of cortical representation in IEHR. In IAHR, motor cortex was reorganized to juxtalesional areas, including the premotor cortex, leg motor cortex and/or sensory cortex, and thus was not localized within the homologous APB motor area in the non-lesioned hemisphere.

### Hand function

Hand function ranged from non-functional to functional in both the developmental and acquired injury groups (Table 2). Of the developmental lesion group, eight had non-functional grasp function whilst 10 had functional grasp function. In the acquired injury group, 24 had non-functional grasp function, whilst eight had functional grasp function (Table 2). Of the IEHR group, 19 had non-functional grasp function, whilst two had functional grasp function. Of the IAHR group, 10 had non-functional grasp function, whilst 15 had functional grasp function (Table 3). *χ*^2^ testing did not find a significant effect of lesion size on resulting hand function (*P* = 0.2); however, only incomplete lesions (i.e. lesions with motor cortex involvement of <50% or >50% but incomplete) were included in this analysis, as complete motor cortex lesions with available hand function data (*N* = 6) always resulted in non-functional hand ability. Fisher’s exact test found a significant effect of injury aetiology on functional outcome, indicating poorer functional outcome for acquired injuries (*P* = 0.02) compared to developmental lesions. Fisher’s exact test for an effect of reorganization type on functional outcome also found a significant effect, indicating poorer functional outcome following IEHR (*P* < 0.001) when compared with IAHR.

### White matter tracts

**Figure 4:**
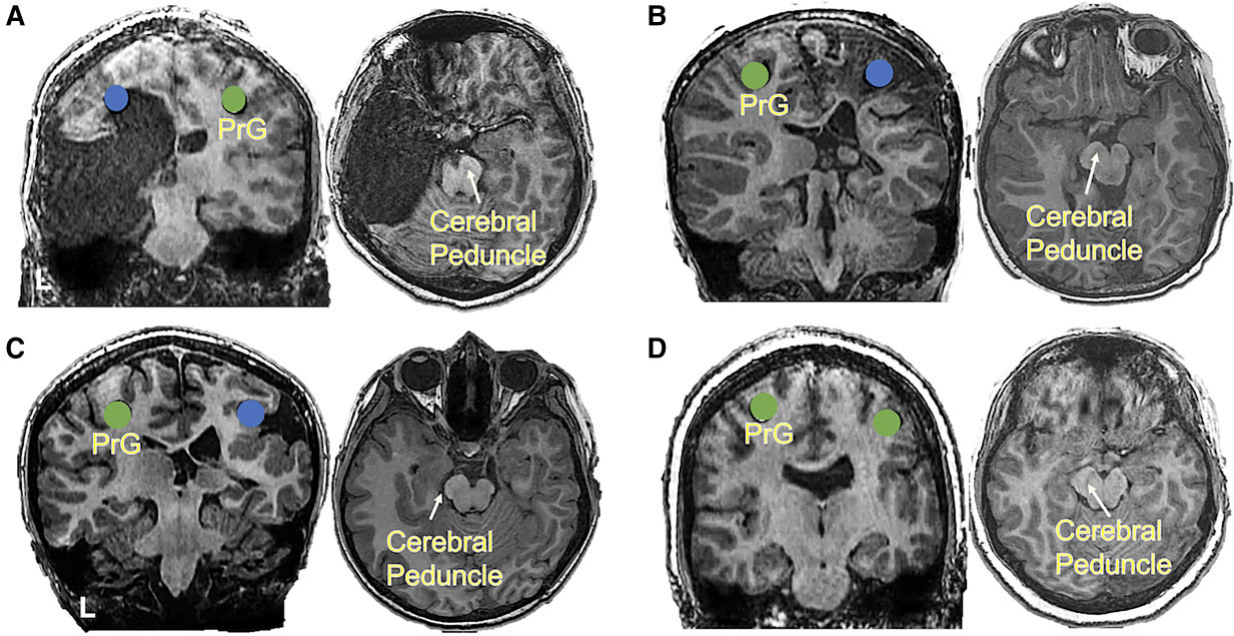
**Case examples of white matter tract viability.** PrG, precentral gyrus; green circles, viable motor cortex; blue circles, homologous inviable motor cortex. (**A**) An 18-year-old female suffering from left hemisphere traumatic brain injury sustained before 2 years of age and resulting IEHR with no remaining left hemisphere motor cortex function. The coronal view of the MRI shows no connection between the cortex and descending spinal tract and the axial view demonstrates the asymmetry of the cerebral peduncle. (**B**) A 6-year-old male suffering from right hemisphere traumatic brain injury before 2 years with no remaining right hemisphere motor cortex function and subsequent IEHR. Coronal MRI demonstrates sparse white matter within the right hemisphere; however, apparent lack of descending white matter and the presence of severely damaged right motor cortex results in non-viable cortex to white matter connectivity. The axial MRI demonstrates significant asymmetry of the cerebral peduncle. (**C**) An 11-year-old male with extensive right hemisphere polymicrogyria, especially affecting the motor cortex. This patient demonstrates a rare case of a developmental disorder resulting in subsequent IEHR. (**D**) A 19-year-old female with focal polymicrogyria who displays resulting IAHR. This patient’s white matter tracts appear to be intact in both hemispheres, demonstrate a high degree of symmetry with respect to descending white matter volume.

In patients exhibiting IEHR, white matter tracts descending from the motor cortex were generally qualitatively non-viable. Additionally, significant asymmetry of pyramidal tracts between affected and unaffected hemispheres at the level of the cerebral peduncle was observed, with the pyramidal tracts in the intact hemisphere being much larger (Fig. 4A–C). The unaffected hemisphere also demonstrated more white matter underneath the motor cortex. In patients exhibiting IAHR, white matter tracts were qualitatively viable, and sufficient symmetry was observed at the subcortical cerebral peduncle level (Fig. 4D). In the four patients who exhibited IAHR with the acquisition of injury before the age of 2 years, the residual cortex connecting to white matter remained, although asymmetry of white matter density between hemispheres was still observed. Resulting motor functions were severely impaired for this sub-group.

## Discussion

The present study demonstrates novel information regarding developmental motor plasticity obtained through the use of an unprecedented clinical cohort size (*N* *=* 52) analysed with TMS. The results from the current study concur with the currently accepted developmental model of the regression of ipsilateral corticospinal projections by 24 months of postnatal development if significant acquired motor cortex injury does not occur. This is supported by our data, where 67% of children (20 of 30) who presented with an acquired injury before 2 years of age demonstrated IEHR. Although not statistically tested due to the small sample size of this sub-group, all patients with injury after the age of 2 years displayed IAHR (Supplementary Table 1). We also found that motor cortex COG distances in patients with IEHR were significantly shorter (*P* < 0.001) than those with IAHR, indicating bilaterally shared cortex in the case of IEHR. This highly significant difference is likely driven by our exclusion criteria, as only aberrant motor representations were included in the IAHR group; however, the significant amount of overlap between ipsilaterally and contralaterally projecting COGs observed in IEHR (Figs. 2B and 3A) indicates that cortical real estate is likely shared. Furthermore, the results demonstrate that lesion aetiology has an explicit effect on the resulting type of cortical reorganization. That is, acquired injuries are significantly more likely to induce IEHR than developmental disorders (*P* < 0.001). Finally, IEHR and acquired injuries resulted in significantly poorer hand function than IAHR and developmental disorders, respectively (*P *< 0.001 and *P *= 0.02).

The results from this study, namely the finding that cortical real estate is likely shared in the case of IEHR, yields insight into the mechanistic nature of IEHR. Descending corticospinal axon development has been repeatedly shown to be activity dependent.^10,11,22^ That is, descending corticospinal axon connections are enhanced by actual axon use. Without axonal activity, the ipsilateral corticospinal axons regress.^22^ In the case of complete unilateral motor cortex injury early in life, the use of the injured hemisphere’s descending tracts becomes impossible. Therefore, the inherent activity-dependent competition favours the viable ipsilateral projections of the unaffected hemisphere, as contralateral projections are rendered obsolete. In the case of unilateral motor cortex injury involving descending white matter tracts, the activity-dependent nature of development coupled with the competition-free environment for contralesional ipsilateral projections most likely causes increased ipsilateral projection development, ultimately resulting in IEHR. Our results add to the understanding of this mechanism in the sense that it has now been shown that the cortical origins of ipsilateral and contralateral projections appear to be shared following IEHR. It can thus be inferred that the existing ipsilateral projections occurring early in development at least partially share descending corticospinal axons with contralateral projections. To establish bilateral alpha-motoneuron synapses following unilateral motor cortex injury early in life, increased ipsilateral axon activity likely promotes axonal sprouting in the distal muscles ipsilateral to the unaffected hemisphere, concurrent with the earlier postulated mechanistic models and primary findings.^21,26,29^ This axonal sprouting establishes more extensive and enhanced connections, resulting in bilateral motor control of single hemispheric origin. Findings from our qualitative white matter tract analysis appear to support this notion. Figure 4 demonstrates that IEHR subjects show descending white matter proliferation of the unaffected hemisphere, especially when the contralateral lesioned cortex is unable to produce viable connectivity. The specifics of ipsilaterally projecting corticospinal networks present during development need further study, as their exact principles of connectivity are largely unknown. Diffusion tensor imaging would provide pertinent information towards a better understanding of mechanistic white matter connectivity following IEHR.

Contralateral and ipsilateral projections appear to share cortical area, implying that ipsilateral and contralateral corticospinal projections stem from the same axons; however, bilateral proliferation by way of axonal sprouting over time must eventually distinguish the laterality of muscle control.^44^ This rewiring potentially uncrosses normally crossed neuronal pathways, as well as increases the total number of corticospinal projections.^12,22,45^ The way in which cortical activity modulates lateralized muscle control is largely unknown. In healthy controls, differentiation between ipsilateral and contralateral descending pathways was implicated through differing corticomotor latencies.^45^ Our population, however, displayed similar latencies for ipsilateral and contralateral innervation, suggesting that injury causes differentiation through axonal sprouting of the same neurons. Thus, mediation of laterality control after the injury is likely to involve different mechanisms. Our finding that rMTs were significantly higher for ipsilateral than contralateral hand muscles (Table 3) indicates modulation of cortical excitability as a potential mechanism for differentiable control. This may involve enhancement of ipsilateral inhibitory control present in normal populations,^46^ but this mechanism has yet to be examined in a patient population. Additionally, our cohort included 47 patients with a prior history of epilepsy and 44 patients with at least one AED prescription (see Supplementary Tables 1 and 2 for more detailed information regarding the history of epilepsy within our cohort). Epilepsy and AED have been shown to be associated with dysregulated cortical excitability, especially within Rolandic cortices.^47–49^ This introduces a potential confounding variable within the proposed mechanism, as dysregulated cortical excitability may underlie the differences in rMT between ipsilateral and contralateral innervation. However, the within-subjects design of this study was meant to control for this confound, as subjects acted as their own control. In other words, the same cortical area was analysed against itself when testing for rMT differences, increasing the likelihood that excitability differences between ipsilateral and contralateral innervation are due to the neurophysiology of IEHR rather than comorbid epilepsy. Furthermore, we found no across-group differences in either the number of patients on AEDs or the number of AEDs prescribed, suggesting that our findings are not likely influenced by AEDs. Measuring the factors governing intracortical inhibition and intracortical facilitation in a patient population similar to ours (i.e. with comorbid epilepsy and IEHR or IAHR) in a more purposeful and directed manner may provide insight into the proposed mechanism of differentiating between the laterality of motor control in the event of IEHR.

Injuries to the motor cortex occurring after ipsilateral projection regression (after 2 years of age) almost always induce IAHR. Our finding that motor cortex COG reorganizes to new cortical area during IAHR confirms the implications made by other studies. Motor cortex reorganization to juxtalesional cortex is observed in Fig. 3. This study supports the claim that in the case of IAHR, parallel motor pathways originating from juxtalesional cortices outside of the primary motor cortex such as the premotor cortex, supplementary motor area and cingulate cortex may accept cortical motor control, aiding in functional recovery.^28,44,50^ Each of the aforementioned cortical areas contain somatotopic representations and all contribute to the pyramidal tract.^24^ These immediate corticomotor changes are most likely modulated by latent synapse unmasking through alterations in GABAergic inhibition, which are then reinforced through activity-dependent reinforcement by way of axonal regeneration, long-term potentiation and axonal sprouting.^2,29^ Additionally, the way in which IAHR eligibility determinations were made (i.e. motor reorganization outside of the normally accepted deviation for primary hand motor cortex)^33–39^ ensured that IAHR, as observed within our cohort, exemplified significant reorganization and juxtalesional motor acquisition similar to that of the aforementioned studies.^28,44,50^

In order for juxtalesional motor function acquisition to occur, cortical real estate must be readily available. Therefore, lesion size plays an important role in the resulting reorganization type. For example, it has been observed that large and incomplete motor cortex injuries resulted in IAHR, whilst large and complete motor cortex injuries showed a higher prevalence of IEHR.^20^ These observations are corrobarated in our study, as every patient with a complete motor cortex injury (*N* = 8) exhibited IEHR, whilst large and incomplete injuries (i.e. >50% but incomplete motor cortex involvement) versus small and incomplete injuries (i.e. <50% motor cortex involvement) had no effect on the resulting type of reorganization (*P* = 0.611). Additionally, the eight patients with injury occurring before the age of 2 years and consequent IAHR demonstrated residual cortex connected to descending white matter tracts following their injury; however, each of these individuals demonstrated severe motor impairments with poor functional recovery. Although lesion size does play a significant role in reorganization type, these results suggest that other factors, such as lesion aetiology and lesion timing, most likely play a more definitive role in the resulting implications of corticomotor reorganization.

Our study found a statistically significant effect of lesion aetiology on resulting corticomotor reorganization, suggesting that acquired brain injuries are far more likely to induce IEHR, whilst developmental brain disorders seem to exhibit a robust tendency to maintain normal hemispheric motor control. Nevertheless, in our study, 3 of the 19 patients presenting with developmental lesion exhibited IEHR (example in Figs. 2B and 4C). This is in parallel to Maegaki *et al*.’s report^29^ of a 13-year-old female presenting with extensive unilateral cortical dysplasia and mild hemiparesis exhibiting IEHR, a rare case of cortical dysplasia in which the motor areas failed to develop to functional levels. In these instances, the lack of descending white matter tract integrity due to pervasive developmental lesion is most likely the driving factor for IEHR. The overwhelming majority of children with developmental disorders in our study exhibited IAHR, demonstrating functional dysplastic cortex located within the motor areas. In concurrence with these findings, fMRI activation patterns in a recent study of two children with rolandic-area focal cortical dysplasia type IIb demonstrated functional dysplastic cortex, along with IAHR following surgical lesionectomy.^27^ The robust maintenance of normal hemispheric motor control demonstrated throughout the majority of developmental disorder cases in our study and others suggest at least a partial functional role of motor cortex localized within the dysplastic lesion.

When considering the pathologies of the various developmental disorders present in this study, robust maintenance of normal hemispheric motor control appears intuitive. The three main developmental disorders present in this study—cortical dysplasia, polymicrogyria and grey matter heterotopia—all result from abnormal neuronal migration during development.^51,52^ Neurodevelopmental disorders involving neuronal migration abnormalities create fundamentally different anatomical implications than acquired injuries. Acquired injuries ensue blunt damage to key neural structures involved in motor control; developmental disorders, however, affect localization of corticomotor representation and its degree of functionality through abnormal neuronal migration. Specifically, in the case of polymicrogyria, the connection between corticomotor representation and underlying white matter, although altered, is usually not completely severed.^52^ This maintenance of the cortex to white matter tract connections indicates at least partial motor control from the affected cortex. In the case of acquired injuries, especially very early on in development, white matter tracts and/or their connections to the cortex can be rendered completely non-viable. Figure 4 investigates this phenomenon in depth. Descending white matter tracts of IEHR patients were universally non-viable, as determined by the lack of MEP from the affected hemispheres, and significant asymmetry of white matter was observed in the cerebral peduncle (Fig. 4A and B). Taken together, the non-viable nature of the white matter tracts and functional consequences are consistent with the occurrence of IEHR due to the lack of viable cortex–white matter connection in the affected hemisphere. The asymmetry of the cerebral peduncle implies action-dependent development of descending ipsilateral motor neurons from the unaffected cortex. In contrast, Fig. 4D shows a patient presenting with right hemispheric focal polymicrogyria. As is evident in this patient’s motor map (Fig. 3A), the primary hand motor cortex of the right hemisphere has localized directly over the area of polymicrogyria. Furthermore, this patient’s dysgenic motor cortex maintained functional capability, although fine motor functional deficits were observed. A rare instance of IEHR pattern of reorganization in a patient with extensive polymicrogyria is shown in Fig. 4C. In this case, the asymmetry of the cerebral peduncle appears similar to the cases in both Fig. 4A and B. Thus, this case appears consistent with activity-dependent axonal sprouting of the viable hemisphere due to the severe nature of this patient’s polymicrogyria producing non-viable cortex to white matter tract connections within the affected hemisphere. The two cases show how acquired injuries alter development through blunt force, whilst developmental disorders are more likely to maintain partial function. Ultimately, this study’s findings indicate that developmental disorders of rolandic areas do not typically inhibit hemispheric competition enough to sufficiently recruit consistent activity of contralesional ipsilateral projections, whilst the damage caused by acquired injuries is more likely to irreversibly damage the descending cortex to white matter projections. This corroborates previously held notions^10^ and is also concurrent with others’ findings regarding the importance of white matter integrity and associated reorganization.^19^

The functional relevance of IAHR versus IEHR is largely unknown. Previous reports indicate that patients with IEHR had variable functional outcomes.^17,53^ Some authors have found a positive correlation between ipsilateral MEPs and motor recovery, others a negative one.^54–56^ When associated with recovery, the ipsilateral MEPs had low excitability thresholds and large amplitudes; when associated with poorer outcomes, only low amplitude MEPs were elicited at high-stimulation intensities.^53,54^ However, it remains unclear if clinical, pathophysiological or methodological differences are responsible for the contrasting observations. In our study, IEHR and acquired injuries were independently associated with poor functional outcome, which is consistent with others.^19^ Using our results and building off of previous studies,^11,19,26,57^ we propose that acquired injuries, especially those severely affecting descending white matter tract integrity, significantly cause IEHR, which ultimately leads to poor functional outcome due to the difficulty of simultaneously modulating bilateral representation from a common cortical location.

In patients with early hemispherectomy, TMS of the intact hemisphere produced ipsilateral MEPs at latencies similar to contralateral MEPs, with higher amplitudes in proximal rather than distal muscles.^24,30^ Patients with late hemispherectomy had ipsilateral MEPs of longer latencies and lower amplitudes with poorer outcome compared to early hemispherectomy patients.^24,30^ In our study, 5 of the 52 patients underwent either complete or partial hemispherectomy at some point during their treatment. Each patient demonstrated functional improvement following the procedure. Furthermore, each patient underwent the procedure early enough (i.e. before 2 years of age) that the remaining hemisphere was able to acquire bilateral motor function. The beneficial results of hemispherectomy suggest a release of inhibitory or degrading functions from the affected hemisphere, which in turns leads to improved functional recovery.^20,58^ Thus, in the case of IEHR and poor motor function, hemispherectomy may be a viable treatment for motor function recovery in addition to the elimination of seizures.

Additionally, the location of somatosensory cortex following corticomotor reorganization may play a role in the resulting functional outcome. Some have found that in patients with substantial sensorimotor lesions early in life, somatosensory cortex exhibits a robust tendency to maintain hemispheric orientation, even if motor function is reorganized to the opposite hemisphere.^17,59^ The same investigators also found that when motor and sensory function are dissociated, the quality of motor function is usually more affected, irrespective of the degree of sensory impairment.^17,59^ The link between sensory reorganization and motor reorganization needs further study, as the implications of differing sensory and motor reorganization patterns and their effect on functional recovery are currently not well understood.

Collectively, the novel information gained from this study regarding the underlying neurophysiological principles governing corticomotor reorganization may be useful in developing new and/or improved therapeutic techniques to assist in the recovery of motor function. For instance, constraint-induced movement therapy (CIMT) has been repeatedly shown to facilitate beneficial neuroplastic changes following unilateral motor cortex injury. Interestingly, CIMT has been shown to elicit functional outcomes linked specifically to the type of reorganization present and the timing of therapeutic intervention relative to an injury.^60–62^ Overall, this suggests that knowledge of basic neurophysiological principles regarding corticomotor reorganization type (IAHR or IEHR) is critical to facilitate the optimal level of functional recovery.

Finally, our results provide evidence for the safety and efficacy of TMS in localizing eloquent cortex. Studies comparing TMS to various neuroimaging modalities (e.g. PET, functional MRI and direct cortical stimulation) have revealed substantive TMS accuracy for such purposes, especially in patients with epilepsy, brain tumour and other neurological diseases.^31,32,63–70^ Accurate localization of motor cortex is challenging in young and developmentally delayed patients, and many modalities require substantial compliance, natural sleep or sedation. Unlike other methods, TMS is well suited for mapping the motor cortices in children and patients with cerebral palsy or developmental delay. Because TMS directly activates the target neurons and corticospinal tract, it can identify the presence or absence of motor cortex regardless of the patient’s motor function or ability, making it uniquely suited for use in those with hemiplegia or paresis. In this cohort, TMS is also useful in mapping treatment-induced changes in motor organization. Our study serves to add to the literature demonstrating the efficacy and safety of TMS in these populations, as well as its utility in studying the impaired motor system.

### Limitations

Due to the retrospective nature of this study, it was at times difficult to find clear measures on each patient. This difficulty stemmed from the broad age range of patients and clinically completed neuropsychological evaluations, resulting in discontinuity of motor tests performed. Ideally, a prospective study would delineate age-specific motor tests for patients with corticomotor reorganization, in order to better compare functional recovery across reorganization types. Another limitation to this study was our inability to compare corticomotor reorganization results across brain imaging modalities. Although TMS has been repeatedly shown to have high accuracy rates in comparison to other neuroimaging modalities, within-subject corticomotor representation accuracy would have benefited from the convergence of multi-modal brain imaging. More generally, retrospective chart review studies have inherent disadvantages, as repeated hospital visits result in data misinterpretation and diagnostic changes over years of evolving medical records. Future prospective longitudinal studies involving corticomotor reorganization, functional outcomes and therapeutic techniques catered to specific neurophysiological changes are needed. Finally, since this study is the first known study of its kind revealing the potential role of injury aetiology in resulting corticomotor reorganization and the nature of IEHR to share cortical real estate, studies seeking to replicate these results are critical.

## Conclusion

The present study examined a largely paediatric clinical cohort of unprecedented size and provided novel data regarding the basic underlying neurophysiological mechanisms of corticomotor reorganization. Key findings included that acquired injuries are much more likely to cause IEHR than IAHR due to the pathological nature of each lesion and that IEHR results in shared cortical representation of ipsilateral and contralateral muscles. Furthermore, IEHR and acquired injuries, respectively, were shown to produce poorer functional motor outcomes. These findings will aid in refining therapeutic techniques using exact neuroplastic principles to optimize functional outcome following injury to the motor cortex in the developing brain.

## Supplementary Material

fcab300_Supplementary_Data

## Abbreviations

APB =: abductor pollicis brevis
CIMT =: constraint-induced movement therapy
COG =: centre of gravity
IAHR =: intrahemispheric reorganization
IEHR =: interhemispheric reorganization
MSO =: maximum stimulator output
MEP =: motor evoked potential
rMT =: resting motor threshold
TMS =: transcranial magnetic stimulation

## References

[1] Binder E, Leimbach M, Pool E-M, et al Cortical reorganization after motor stroke: A pilot study on differences between the upper and lower limbs. Hum Brain Mapp. 2021;42:1013–1033. 33165996 10.1002/hbm.25275 PMC7856649

[2] Chen R, Cohen LG, Hallett M. Nervous system reorganization following injury. Neuroscience. 2002;111(4):761–773. 12031403 10.1016/s0306-4522(02)00025-8

[3] Pascual-Leone A, Amedi A, Fregni F, Merabet LB. The plastic human brain cortex. Annu Rev Neurosci. 2005;28(1):377–401. 16022601 10.1146/annurev.neuro.27.070203.144216

[4] Choi JT, Vining EPG, Mori S, Bastian AJ. Sensorimotor function and sensorimotor tracts after hemispherectomy. Neuropsychologia. 2010;48(5):1192–1199. 20018199 10.1016/j.neuropsychologia.2009.12.013 PMC2847436

[5] Dennis M. Margaret Kennard (1899–1975): Not a ‘Principle’ of brain plasticity but a founding mother of developmental neuropsychology. Cortex. 2010;46(8):1043–1059. 20079891 10.1016/j.cortex.2009.10.008 PMC2907425

[6] Krägeloh-Mann I, Lidzba K, Pavlova M, Wilke M, Staudt M. Plasticity during early brain development is determined by ontogenetic potential. Neuropediatrics. 2017;48(2):66–71. 28282668 10.1055/s-0037-1599234

[7] Staudt M, Grodd W, Gerloff C, Erb M, Stitz J, Krägeloh-Mann I. Two types of ipsilateral reorganization in congenital hemiparesis: A TMS and fMRI study. Brain. 2002;125(10):2222–2237. 12244080 10.1093/brain/awf227

[8] Inguaggiato E, Sgandurra G, Perazza S, Guzzetta A, Cioni G. Brain reorganization following intervention in children with congenital hemiplegia: A systematic review. Neural Plast. 2013;2013:1–8. 10.1155/2013/356275 PMC3866714 24367726

[9] Ismail FY, Fatemi A, Johnston MV. Cerebral plasticity: Windows of opportunity in the developing brain. Eur J Paediatr Neurol. 2017;21(1):23–48. 27567276 10.1016/j.ejpn.2016.07.007

[10] Eyre JA, Taylor JP, Villagra F, Smith M, Miller S. Evidence of activity-dependent withdrawal of corticospinal projections during human development. Neurology. 2001;57:1543–1554. 11706088 10.1212/wnl.57.9.1543

[11] Eyre JA. Development and plasticity of the corticospinal system in man. Neural Plast. 2003;10(1–2):93–106. 14640311 10.1155/NP.2003.93 PMC2565418

[12] Sebastianelli L, Versace V, Taylor A, et al Functional reorganization after hemispherectomy in humans and animal models: What can we learn about the brain’s resilience to extensive unilateral lesions? Brain Res Bull. 2017;131:156–167. 28414105 10.1016/j.brainresbull.2017.04.005

[13] Staudt M. (Re-)organization of the developing human brain following periventricular white matter lesions. Neurosci Biobehav Rev. 2007;31(8):1150–1156. 17624432 10.1016/j.neubiorev.2007.05.005

[14] Staudt M. Reorganization after pre- and perinatal brain lesions. J Anat. 2010;217(4):469–474. 20649910 10.1111/j.1469-7580.2010.01262.x PMC2992421

[15] Narayana S, Gibbs SK, Fulton SP, et al Clinical utility of transcranial magnetic stimulation (TMS) in the presurgical evaluation of motor, speech, and language functions in young children with refractory epilepsy or brain tumor: Preliminary evidence. Front Neurol. 2021;12:650830. 34093397 10.3389/fneur.2021.650830 PMC8170483

[16] Fiori S, Guzzetta A. Plasticity following early-life brain injury: Insights from quantitative MRI. Semin Perinatol. 2015;39(2):141–146. 25813668 10.1053/j.semperi.2015.01.007

[17] Gupta D, Barachant A, Gordon AM, et al Effect of sensory and motor connectivity on hand function in pediatric hemiplegia. Ann Neurol. 2017;82(5):766–780. 29034483 10.1002/ana.25080 PMC5708868

[18] Payne BR, Lomber SG. Reconstructing functional systems after lesions of cerebral cortex. Nat Rev Neurosci. 2001;2(12):911–919. 11733798 10.1038/35104085

[19] Holmström L, Vollmer B, Tedroff K, et al Hand function in relation to brain lesions and corticomotor-projection pattern in children with unilateral cerebral palsy. Dev Med Child Neurol. 2010;52(2):145–152. 19807768 10.1111/j.1469-8749.2009.03496.x

[20] Irle E. Lesion size and recovery of function: Some new perspectives. Brain Res Rev. 1987;12(3):307–320. 10.1016/0165-0173(87)90003-8 3111643

[21] Maegaki Y, Maeoka Y, Ishii S, et al Mechanisms of central motor reorganization in pediatric hemiplegic patients. Neuropediatrics. 1997;28(3):168–174. 9266555 10.1055/s-2007-973695

[22] Martin JH, Kably B, Hacking A. Activity-dependent development of cortical axon terminations in the spinal cord and brain stem. Exp Brain Res. 1999;125(2):184–199. 10204771 10.1007/s002210050673

[23] Staudt M, Gerloff C, Grodd W, Holthausen H, Niemann G, Krägeloh-Mann I. Reorganization in congenital hemiparesis acquired at different gestational ages. Ann Neurol. 2004;56(6):854–863. 15562409 10.1002/ana.20297

[24] Hallett M. Plasticity of the human motor cortex and recovery from stroke. Brain Res Rev. 2001;36(2–3):169–174. 11690613 10.1016/s0165-0173(01)00092-3

[25] Jankowska E, Edgley SA. How can corticospinal tract neurons contribute to ipsilateral movements? A question with implications for recovery of motor functions. Neuroscientist. 2006;12(1):67–79. 16394194 10.1177/1073858405283392 PMC1890027

[26] Reddy H, De Stefano N, Mortilla M, Federico A, Matthews PM. Functional reorganization of motor cortex increases with greater axonal injury from CADASIL. Stroke. 2002;33(2):502–508. 11823660 10.1161/hs0202.103337

[27] Barba C, Montanaro D, Frijia F, et al Focal cortical dysplasia type IIb in the rolandic cortex: Functional reorganization after early surgery documented by passive task functional MRI. Epilepsia. 2012;53(8):e141–e145. 22686520 10.1111/j.1528-1167.2012.03524.x

[28] Duffau H. Acute functional reorganisation of the human motor cortex during resection of central lesions: A study using intraoperative brain mapping. J Neurol Neurosurg Psychiatry 2001;70(4):506–513. 11254775 10.1136/jnnp.70.4.506 PMC1737325

[29] Maegaki Y, Yamamoto T, Takeshita K. Plasticity of central motor and sensory pathways in a case of unilateral extensive cortical dysplasia: Investigation of magnetic resonance imaging, transcranial magnetic stimulation, and short-latency somatosensory evoked potentials. Neurology. 1995;45(12):2255–2261. 8848203 10.1212/wnl.45.12.2255

[30] Benecke R, Meyer B-U, Freund H-J. Reorganisation of descending motor pathways in patients after hemispherectomy and severe hemispheric lesions demonstrated by magnetic brain stimulation. Exp Brain Res. 1991;83(2):419–426. 2022247 10.1007/BF00231167

[31] Narayana S, Mudigoudar B, Babajani-Feremi A, Choudhri AF, Boop FA. Successful motor mapping with transcranial magnetic stimulation in an infant: A case report. Neurology. 2017;89(20):2115–2117. 29021352 10.1212/WNL.0000000000004650

[32] Narayana S, Papanicolaou AC, McGregor A, Boop FA, Wheless JW. Clinical applications of transcranial magnetic stimulation in pediatric neurology. J Child Neurol. 2015;30(9):1111–1124. 25342309 10.1177/0883073814553274

[33] Ahdab R, Ayache SS, Brugières P, Goujon C, Lefaucheur JP. Comparison of “standard” and “navigated” procedures of TMS coil positioning over motor, premotor and prefrontal targets in patients with chronic pain and depression. Neurophysiol Clin Neurophysiol. 2010;40(1):27–36. 10.1016/j.neucli.2010.01.001 20230933

[34] Ahdab R, Ayache SS, Brugières P, Farhat WH, Lefaucheur J-P. The hand motor hotspot is not always located in the hand knob: A neuronavigated transcranial magnetic stimulation study. Brain Topogr. 2016;29(4):590–597. 26980192 10.1007/s10548-016-0486-2

[35] Kantelhardt SR, Fadini T, Finke M, et al Robot-assisted image-guided transcranial magnetic stimulation for somatotopic mapping of the motor cortex: A clinical pilot study. Acta Neurochir (Wien). 2010;152(2):333–343. 19943069 10.1007/s00701-009-0565-1 PMC2815301

[36] Niskanen E, Julkunen P, Säisänen L, Vanninen R, Karjalainen P, Könönen M. Group-level variations in motor representation areas of thenar and anterior tibial muscles: Navigated transcranial magnetic stimulation study. Hum Brain Mapp. 2010;31:1272–1280. 20082330 10.1002/hbm.20942 PMC6870707

[37] Diekhoff S, Uludağ K, Sparing R, et al Functional localization in the human brain: Gradient-echo, spin-echo, and arterial spin-labeling fMRI compared with neuronavigated TMS. Hum Brain Mapp. 2011;32(3):341–357. 20533563 10.1002/hbm.21024 PMC6870385

[38] Julkunen P, Ruohonen J, Sääskilahti S, Säisänen L, Karhu J. Threshold curves for transcranial magnetic stimulation to improve reliability of motor pathway status assessment. Clin Neurophysiol. 2010;122:975–983. 10.1016/j.clinph.2010.09.005 20888291

[39] Roux F-E, Niare M, Charni S, Giussani C, Durand J-B. Functional architecture of the motor homunculus detected by electrostimulation. J Physiol. 2020;598(23):5487–5504. 32857862 10.1113/JP280156

[40] Rossi S, Antal A, Bestmann S, et al Safety and recommendations for TMS use in healthy subjects and patient populations, with updates on training, ethical and regulatory issues: Expert Guidelines. Clin Neurophysiol. 2021;132(1):269–306. 33243615 10.1016/j.clinph.2020.10.003 PMC9094636

[41] Wilson SA, Thickbroom GW, Mastaglia FL. Transcranial magnetic stimulation mapping of the motor cortex in normal subjects. J Neurol Sci. 1993;118(2):134–144. 8229061 10.1016/0022-510x(93)90102-5

[42] Karl A, Birbaumer N, Lutzenberger W, Cohen LG, Flor H. Reorganization of motor and somatosensory cortex in upper extremity amputees with phantom limb pain. J Neurosci. 2001;21(10):3609–3618. 11331390 10.1523/JNEUROSCI.21-10-03609.2001 PMC6762494

[43] Kwon HG, Hong JH, Jang SH. Anatomic location and somatotopic arrangement of the corticospinal tract at the cerebral peduncle in the human brain. Am J Neuroradiol. 2011;32(11):2116–2119. 21903908 10.3174/ajnr.A2660 PMC7964417

[44] Fries W, Danek A, Scheidtmann K, Hamburger C. Motor recovery following capsular stroke: Role of descending pathways from multiple motor areas. Brain. 1993;116(2):369–382. 8461971 10.1093/brain/116.2.369

[45] Ziemann U, Ishii K, Borgheresi A, et al Dissociation of the pathways mediating ipsilateral and contralateral motor-evoked potentials in human hand and arm muscles. J Physiol. 1999;518(3):895–906. 10420023 10.1111/j.1469-7793.1999.0895p.x PMC2269467

[46] Chen R, Yung D, Li J-Y. Organization of ipsilateral excitatory and inhibitory pathways in the human motor cortex. J Neurophysiol. 2003;89(3):1256–1264. 12611955 10.1152/jn.00950.2002

[47] Pawley AD, Chowdhury FA, Tangwiriyasakul C, et al Cortical excitability correlates with seizure control and epilepsy duration in chronic epilepsy. Ann Clin Transl Neurol. 2017;4(2):87–97. 28168208 10.1002/acn3.383 PMC5288462

[48] Andreasson A-C, Sigurdsson GV, Pegenius G, Thordstein M, Hallböök T. Cortical excitability measured with transcranial magnetic stimulation in children with epilepsy before and after antiepileptic drugs. Dev Med Child Neurol. 2020;62(7):793–798. 32064586 10.1111/dmcn.14490

[49] Hamed SA, Tohamy AM. Mohamed KO, el Mageed Abd el Zaher MA. The effect of epilepsy and antiepileptic drugs on cortical motor excitability in patients with temporal lobe epilepsy. Clin Neuropharmacol. 2020;43(6):175–184. 32969972 10.1097/WNF.0000000000000412

[50] Dum R, Strick P. The origin of corticospinal projections from the premotor areas in the frontal lobe. J Neurosci. 1991;11(3):667–689. 1705965 10.1523/JNEUROSCI.11-03-00667.1991 PMC6575356

[51] Desikan RS, Barkovich AJ. Malformations of cortical development. Ann Neurol. 2016;80(6):797–810. 27862206 10.1002/ana.24793 PMC5177533

[52] Squier W, Jansen A. Polymicrogyria: Pathology, fetal origins and mechanisms. Acta Neuropathol Commun. 2014;2(1):80. 25047116 10.1186/s40478-014-0080-3 PMC4149230

[53] Caramia MD, Iani C, Bernardi G. Cerebral plasticity after stroke as revealed by ipsilateral responses to magnetic stimulation. NeuroReport. 1996;7(11):1756–1760. 8905658 10.1097/00001756-199607290-00012

[54] Turton A, Wroe S, Trepte N, Fraser C, Lemon RN. Contralateral and ipsilateral EMG responses to transcranial magnetic stimulation during recovery of arm and hand function after stroke. Electroencephalogr Clin Neurophysiol Mot Control. 1996;101(4):316–328. 10.1016/0924-980x(96)95560-5 8761041

[55] Hendricks HT, Hageman G, van Limbeek J. Prediction of recovery from upper extremity paralysis after stroke by measuring evoked potentials. Scand J Rehabil Med. 1997;29(3):155–159. 9271149

[56] Netz J. Reorganization of motor output in the non-affected hemisphere after stroke. Brain. 1997;120(9):1579–1586. 9313641 10.1093/brain/120.9.1579

[57] Eyre J. Corticospinal tract development and its plasticity after perinatal injury. Neurosci Biobehav Rev. 2007;31(8):1136–1149. 18053875 10.1016/j.neubiorev.2007.05.011

[58] Chiricozzi F, Chieffo D, Battaglia D, et al Developmental plasticity after right hemispherectomy in an epileptic adolescent with early brain injury. Childs Nerv Syst. 2005;21(11):960–969. 15856259 10.1007/s00381-005-1148-y

[59] Guzzetta A, Bonanni P, Biagi L, et al Reorganisation of the somatosensory system after early brain damage. Clin Neurophysiol. 2007;118(5):1110–1121. 17382585 10.1016/j.clinph.2007.02.014

[60] Walther M, Juenger H, Kuhnke N, et al Motor cortex plasticity in ischemic perinatal stroke: A transcranial magnetic stimulation and functional MRI study. Pediatr Neurol. 2009;41(3):171–178. 19664531 10.1016/j.pediatrneurol.2009.04.006

[61] Matusz PJ, Key AP, Gogliotti S, et al Somatosensory plasticity in pediatric cerebral palsy following constraint-induced movement therapy. Neural Plast. 2018;2018:1–14. 10.1155/2018/1891978 PMC6250030 30532772

[62] Okabe N, Himi N, Nakamura-Maruyama E, et al Very early initiation reduces benefits of poststroke rehabilitation despite increased corticospinal projections. Neurorehabil Neural Repair. 2019;33(7):538–552. 31140375 10.1177/1545968319850132

[63] Wassermann EM, Wang B, Zeffiro TA, et al Locating the motor cortex on the MRI with transcranial magnetic stimulation and PET. NeuroImage. 1996;3(1):1–9. 9345470 10.1006/nimg.1996.0001

[64] Thickbroom GW, Byrnes ML, Mastaglia FL. A model of the effect of MEP amplitude variation on the accuracy of TMS mapping. Clin Neurophysiol. 1999;110(5):941–943. 10400209 10.1016/s1388-2457(98)00080-7

[65] Krieg TD, Salinas FS, Narayana S, Fox PT, Mogul DJ. PET-based confirmation of orientation sensitivity of TMS-induced cortical activation in humans. Brain Stimul. 2013;6(6):898–904. 23827648 10.1016/j.brs.2013.05.007 PMC5293002

[66] Forster M-T, Hattingen E, Senft C, Gasser T, Seifert V, Szelényi A. Navigated transcranial magnetic stimulation and functional magnetic resonance imaging: Advanced adjuncts in preoperative planning for central region tumors. Neurosurgery. 2011;68(5):1317–1325. 21273929 10.1227/NEU.0b013e31820b528c

[67] Uy J, Ridding MC, Miles TS. Stability of maps of human motor cortex made with transcranial magnetic stimulation. Brain Topogr. 14(4):293–297. 12137362 10.1023/a:1015752711146

[68] Mäkelä JP, Vitikainen A-M, Lioumis P, et al Functional plasticity of the motor cortical structures demonstrated by navigated TMS in two patients with epilepsy. Brain Stimul. 2013;6(3):286–291. 22659020 10.1016/j.brs.2012.04.012

[69] Vitikainen A-M, Lioumis P, Paetau R, et al Combined use of non-invasive techniques for improved functional localization for a selected group of epilepsy surgery candidates. NeuroImage. 2009;45(2):342–348. 19159694 10.1016/j.neuroimage.2008.12.026

[70] Vitikainen A-M, Salli E, Lioumis P, Mäkelä JP, Metsähonkala L. Applicability of nTMS in locating the motor cortical representation areas in patients with epilepsy. Acta Neurochir (Wien). 2013;155(3):507–518. 23328919 10.1007/s00701-012-1609-5

