# Supplementary material for: Plasticity in the developing brain: neurophysiological basis for lesion-induced motor reorganization
Source: Brain Commun. 2021 Dec 21;4(1):fcab300. doi: 10.1093/braincomms/fcab300 (PMC8842689; doi:10.1093/braincomms/fcab300)
Supplement: fcab300_Supplementary_Data [file fcab300_Supplementary_Data.pdf]

Supplementary Table I: Clinical Characteristics of Acquired Lesion Group

Patient Number	Sex	Age at TMS (years)	Lesion location and type	Lesion Extent Within Motor Cortex/White Matter Tracts	Type of Reorganization	Reason for Clinical Evaluation/TMS Mapping	Antiepileptic Medications (AEDs)	Grasp Function (functional v. non-functional)
A1	F	1.7	LH Hemimegalencephaly s/p Hemispherectomy at 3 months of age	Complete damage to motor cortex and underlying white matter tracts	IEHR	Diagnostic evaluation of new onset paroxymal events with h/o refractory seizures resolved following left functional hemispherectomy	None	non-functional
A2	F	1.8	LH perinatal stroke with damage to left cerebral peduncle and the left medullary pyramid	Intact motor cortex with extensive damage to underlying white matter tracts	IEHR	Phase I evaluation of intractable symptomatic localization related epilepsy - LH origin	Zonisamide and oxcarbazepine	functional
A3	F	1.9	LH perinatal stroke with cystic encephalomalacia involving the majority of the left cerebral hemisphere	> 50% damage to motor cortex with sparing of medial part of CS and extensive damage to underlying white matter tracts	IEHR	Phase I evaluation of intractable symptomatic localization related epilepsy - LH origin	Lacosamide and levetiracetam	non-functional
A4	M	2.1	LH perinatal stroke with encephalomalacia involving left MCA territory. Marked atrophy of left lentiform nucleus, caudate nucleus, and cerebral peduncle.	< 50% damage to motor cortex with sparing of medial part of CS, extensive damage to underlying white matter tracts	IEHR	Phase I evaluation of intractable symptomatic focal epilepsy - LH origin	Lacosamide, clobazam, and lamotrigine	non-functional
A5	F	3.3	LH perinatal stroke with diffuse white matter volume loss and areas of gliosis involving the left cerebral hemisphere including the precentral and post central gyri. Cystic encephalomalacia of the left putamen and posterior limb of the internal capsule. Marked atrophy of left caudate nucleus, globus pallidus, and left cerebral peduncle.	Intact motor cortex, but extensive damage to underlying white matter tracts	IEHR	Phase I evaluation of intractable symptomatic partial seizures	Levetiracetam	functional
A6	F	6.3	LH perinatal stoke, s/p LH functional hemispherectomy at 1.7 years of age	> 50% damage to motor cortex, sparing lateral parts of CS; extensive damage to underlying white matter tracts	IEHR	Diagnostic evaluation of new paroxysmal events with h/o controlled right hemisphere complex partial seizures, resolved symptomatic partial seizures of left hemisphere origin.	Lacosamide	non-functional
A7	F	6.4	RH atypical teratoid rhabdoid tumor s/p resection of portions of the right insula, lentiform nucleus, and posterior limb of the internal capsule at 15 months	< 50% damage to motor cortex with sparing of lateral part of CS, extensive damage to underlying white matter tracts	IEHR	Diagnostic evaluation of symptomatic partial epilepsy secondary to tumor in remission s/p surgical resection, chemotherapy, and focal radiotherapy.	Oxcarbazepine and valproic acid	non-functional
A8	M	6.5	RH Traumatic Brain Injury at 16 m of age, extensive cystic encephalomalacia involving nearly the entirety of the right frontal lobe.	Complete damage to motor cortex and underlying white matter tracts	IEHR	Diagnostic evaluation of intractable, symptomatic partial onset seizures	Clobazam	non-functional
A9	F	8.2	RH perinatal stroke in right MCA territory with perisylvian cystic encephalomalacia, Marked atrophy of right sided deep gray nuclei, cerebral peduncle, and brainstem	< 50% damage to motor cortex with sparing of lateral part of CS, extensive damage to underlying white matter tracts	IEHR	Diagnostic evaluation of intractable focal epilepsy, RH origin	Zonisamide, lacosamide, and levetiracetam	non-functional

A10	F	9.5	RH Herpes Simplex encephalitis at 20 m of age with extensive encephalomalacia in the right hemisphere, maximal over posterior frontal and parietal cortex. Atrophy of the right posterior limb of the internal capsule, peduncle, and pons; marked decrease in white matter in the right hemisphere	Complete damage to motor cortex and underlying white matter tracts	IEHR	Phase I evaluation of intractable, symptomatic generalized tonic seizures of right hemisphere origin	Topiramate, clobazam, valproic acid, and rufinamide	non-functional
A11	F	9.9	RH Traumatic Brain Injury before 2 y. Extensive encephalomalacia of the right hemisphere and the parasagittal left frontal lobe. Marked atrophy involving the right cerebral peduncle.	Complete damage to motor cortex and underlying white matter tracts	IEHR	Phase I evaluation of intractable symptomatic epilepsy, RH origin	Oxcarbazepine, levetiracetam, and rufinamide	insufficient data
A12	M	10.6	LH perinatal stroke with marked cystic encephalomalacia and volume loss involving the left MCA distribution Atrophy of left cerebral peduncle, pons, and medulla.	> 50% damage to motor cortex, sparing medial parts of CS; extensive damage to underlying white matter tracts	IEHR	Phase I evaluation of symptomatic refractory focal epilepsy, LH origin	Levetiracetam	non-functional
A13	F	12.1	RH perinatal stroke in right MCA territory with cystic encephalomalacia. No internal capsule on the right	> 50% damage to motor cortex, sparing medial parts of CS; extensive damage to underlying white matter tracts	IEHR	Phase I evaluation of intractable, symptomatic partial onset and generalized myoclonic tonic seizures, right hemisphere origin.	None	non-functional
A14	M	12.2	RH perinatal stroke with markedly decreased white matter volume throughout the right cerebral hemisphere with relative preservation of the gray matter volume. He also had marked thinning of the internal capsule in the right hemisphere.	> 50% damage to motor cortex, sparing lateral parts of CS; extensive damage to underlying white matter tracts	IEHR	Phase I evaluation of intractable, symptomatic complex partial seizures of right hemisphere origin.	Oxcarbazepine, clonazepam, and valproic acid	non-functional
A15	F	12.2	RH perinatal stroke with volume loss of the descending fibers in the corticospinal tract and midportion of the posterior limb of the internal capsule, cerebral peduncle, and medulla oblongata.	Cortex along the CS is intact, extensive damage to underlying white matter tracts	IEHR	Phase I evaluation of symptomatic partial seizures of right hemisphere origin.	Oxcarbazepine and clobazam	non-functional
A16	M	12.9	RH perinatal stroke with encephalomalacia in the right posterior frontal, anterior parietal regions; Atrophy of right cerebral peduncle consistent with his early life injury.	< 50% damage to motor cortex with sparing of lateral part of CS, extensive damage to underlying white matter tracts	IEHR	Diagnostic evaluation of intractable, symptomatic partial onset seizures of right frontal lobe origin, and new paroxysmal events	Clonazepam and levetiracetam	non-functional
A17	M	16.3	RH perinatal stroke with extensive gliosis and subsequent resection of RH hemisphere	Complete damage to motor cortex and underlying white matter tracts	IEHR	Phase I evaluation of intractable symptomatic epilepsy. Localized to RH.	None	non-functional
A18	F	16.9	RH perinatal Traumatic Brain Injury s/p hemispherectomy	Complete motor cortex and white matter damage.	IEHR	Evaluation of motor function	Topiramate and oxcarbazepine	non-functional

A19	M	17.2	Bilateral traumatic brain injury, with cystic encephalomalacia involving nearly entirety of both cerebral hemispheres, with relative sparing of both occipital lobes, the inferior and mesial aspects of both temporal lobes, and superior aspect of the right precentral gyrus.	Complete damage of CS with underlying white matter tracts. Motor cortex localized within the right precentral gyrus	IEHR	Phase I evaluation of Intractable symptomatic generalized seizures	Topiramate, clonazepam, clobazam, and perampanel	non-functional
A20	F	18.2	LH Traumatic Brain Injury with evidence of prior left functional hemispherectomy with residual left frontal and occipital parenchyma and rudimentary remnants of the deep gray nuclei.	Complete motor cortex and white matter damage.	IEHR	Evaluation of motor function	Zonisamide, clonazepam, and lamotrigine	non-functional
A21	F	4.4	RH tumor in superior parietal lobule	Intact motor cortex	IAHR- Hand motor cortex Localized within face/mouth motor area.	Presurgical evaluation of partial symptomatic seizures due to Dysembryoplastic neuroepithelial tumor (WHO grade I)	Levetiracetam	functional
A22	F	6.6	LH perinatal stroke with severe gray and white matter volume loss with extensive encephalomalacia/gliosis involving the entire left cerebral hemisphere. Sparing of medial frontal gyrus	> 50% damage to motor cortex, sparing medial parts of CS	IAHR- LH hand motor cortex Localized within leg motor area	Phase I evaluation of intractable simple partial motor seizures and complex partial seizures of left hemisphere origin.	Oxcarbazepine and clobazam	non-functional
A23	F	7.5	LH Dystonic Variant Rasmussen's Encephalitis with left hemisphere atrophy and atrophy of left cerebral peduncle	> 50% damage to motor cortex, sparing lateral parts of CS	IAHR - LH hand motor cortex localized over Postcentral gyrus	Evaluation of motor function	None	non-functional
A24	M	11.5	LH Tumor at 9 m of age: GBM s/p resection and chemotherapy. Resection in left frontal lobe and volume loss of LH.	< 50% damage to motor cortex, sparing medial parts of CS.	IAHR - LH hand motor cortex Localized within leg motor area	Phase I evaluation of intractable symptomatic partial seizures, LH origin	Oxcarbazepine and gabapentin	functional
A25	F	15.8	LH perinatal watershed ischemic injury causing encephalomalacia in the left parietal lobe with ulegyria	Intact motor cortex	IAHR - LH hand motor cortex extends anteriorly into MFG and SFG.	Phase I evaluation of intractable, symptomatic partial onset seizures of left parietal origin	None	functional
A26	M	16.5	LH perinatal MCA stroke with left frontotemporal cystic encephalomalacia, slight atrophy of cerebral peduncle, internal capsule, and lentiform nucleus; part of the left precentral gyrus and postcentral gyrus are preserved.	> 50% damage to motor cortex, sparing medial parts of CS	Mixed: IEHR: Bilateral representation in RH; and IAHR: LH hand motor cortex localized medially within leg motor area	Phase I evaluation of symptomatic partial onset seizures with secondary generalization of left hemisphere origin.	None	non-functional
A27	F	18.9	Bilateral White Matter Damage (R>L) caused by prematurity with moderate to severe parieto-occipital predominant white matter volume loss, and volume loss along the expected course of the corticospinal tracts.	Intact motor cortex in LH and volume loss in RH	IAHR- RH hand motor cortex localized laterally within face/mouth motor area.	Phase I evaluation of intractable, symptomatic generalized and partial seizures	Clonazepam, lamotrigine, and phenobarbital	non-functional
A28	M	3.6	LH parietal lesion suspicious of cortical dysplasia subsequently determined to be a Stage III anaplastic astrocytoma	Intact motor cortex	IAHR - LH hand motor cortex localized over Postcentral gyrus	Phase I evaluation of intractable, symptomatic localization-related epilepsy secondary to LH brain tumor	Oxcarbazepine and levetiracetam	functional

A29	F	4.5	LH Rasmussen's Encephalitis likely onset at 22 months; with severe gray and white matter volume loss with extensive encephalomalacia/gliosis involving the entire left cerebral hemisphere.	> 50% damage to motor cortex, sparing medial parts of CS; extensive damage to underlying white matter tracts	None - LH hand motor cortex not localized. Only contralateral MEPs noted when RH was stimulated.	Phase I evaluation of refractory symptomatic focal and generalized epilepsy secondary to left hemispheric atrophy	Oxcarbazepine and levetiracetam	non-functional
A30	F	8.8	RH Lobectomy at 4 m of age for refractory seizures secondary to TSC. right-sided resection of lentiform nucleus, frontoparietal operculum, posterior aspect of middle frontal gyrus, and inferior midportion of perirolandic cortex with sparing of the parasagittal cortex of the right frontal and parietal lobes. Other stigmata of TSC including tubers and several subendymal nodules along the lateral margin of the body of both lateral ventricles were observed.	> 50% damage to motor cortex, sparing medial parts of CS, extensive damage to underlying white matter tracts	None - RH hand motor cortex not localized. Only contralateral MEPs noted when LH was stimulated.	Phase I evaluation of intractable, symptomatic partial seizures of right hemisphere origin	Valproic acid, phenytoin, and phenobarbital	non-functional
A31	*M*	*12.1*	*RH Tumor; Large frontal subcortical tumor with mass effect and edema; Compression of white matter and ventricles.*	*Complete motor cortex damage*	*IAHR: RH hand motor cortex localized posteriorly over postcentral gyrus and IPL*	*Presurgical evaluation for newly diagnosed Glioblastoma (WHO grade IV). who presented with progressive left sided weakness, headache, vomiting and visual symptoms*	*Levetiracetam*	*Non-functional*
A32	*M*	*14.7*	*RH tumor in posterior frontal lobe and the right inferior parietal lobule. The lesion extends into deep white matter displacing descending fibers of CSP anteromedially; tumor extended into the posterior limb of the internal capsule.*	*> 50% damage to motor cortex, sparing medial parts of CS*	*IAHR - RH hand motor cortex extends anteriorly into MFG and SFG.*	*Presurgical evaluation for recurrence of Progressive anaplastic astrocytoma at age 12.5 with h/o weakness and numbness in left hand*	*Oxcarbazepine and levetiracetam*	*functional*
A33	*F*	*50.0*	*LH Tumor; recurrent Frontal lobe low grade glioma. There is a focal resection cavity seen centered in approximately in the inferior aspect of the left central gyrus. Tumor recurrence along the margins of the prior resection cavity, there is focal involvement in the inferior most aspect of the left precentral gyrus*	*< 50% damage to motor cortex, sparing medial parts of CS.*	*IAHR - LH hand motor cortex extends anteriorly into MFG and SFG.*	*Presurgical evaluation of recurrent tumor with h/o focal motor seizures*	*None*	*functional*

f - female; m - male; LH - left hemisphere; RH - right hemisphere; TSC: Tuberous sclerosis complex; s/p: status post; SMG: suprmarginal gyrus; MFG: middle fronal gyrus; SFG: superior frontal gyrus; IPL: inferior parietal lobule; IFG: inferior frontal gyrus; M1: primary motor cortex

CS: Central sulcus

Italicized: injury after 2 years (A31-A33)

Supplementary Table II: Clinical Characteristics of Developmental Lesion Group

Patient Number	Sex	Age at TMS (years)	Lesion Location and Type	Lesion Extent Within Motor Cortex/White Matter Tracts	Type of Reorganization	Reason for Clinical Evaluation/TMS Mapping	Antiepileptic Medications (AEDs)	Grasp Function (Functional v. Non-functional)
D1	F	6.2	RH extensive dysplasia throughout the underdeveloped cerebral hemisphere, most pronounced in the parietal and occipital lobes with marked white matter volume loss and occipital pachygyria.	Intact motor cortex with extensive damage to underlying white matter tracts	IEHR	Phase I evaluation of intractable symptomatic myoclonic tonic and myotonic tonic-clonic seizures of RH origin	Clonazepam, clobazam, valproic acid, and rufinamide	insufficient data
D2	M	11.7	RH extensive polymicrogyria involving frontal lobe and sylvian fissure. Polymicrogyria extends from the inferior frontal gyrus and superior temporal gyrus to middle and superior frontal gyri. The right precentral and postcentral gyri involved along their inferior lateral aspect but relatively preserved superiorly and along the parasagittal plane.	Normal pattern of CS is lost laterally because of cortical dysplasia	IEHR	Phase I evaluation of partial symptomatic seizures secondary to RH lesion.	Lamotrigine and lorazepam	non-functional
D3	F	16.3	RH dysplastic appearance of the parietal lobe, and insula, with malformation of neuronal migration and post migrational organization, including pachygyria, and band heterotopia.	Greater than 50%, but incomplete motor cortex damage.	IEHR	Phase I evaluation of intractable focal onset seizures secondary to a RH lesion	Oxcarbazepine and levetiracetam	non-functional
D4	M	1.7	RH cortical dysplasia of the posterior parasagittal margin of the superior frontal gyrus	Intact motor cortex	IAHR: RH hand motor cortex localized medially within leg motor area	Phase I evaluation for refractory, symptomatic partial seizures secondary to cortical dysplasia.	Oxcarbazepine and levetiracetam	functional
D5	M	2.9	RH multiple foci of polymicrogyria, subcortical and transmantle heterotopia, with atypical/accessory fissures in occipital, temporal, and posterior parietal lobes, atypical sulci pattern in frontal lobe, atrophy of the thalamus and lentiform nucleus.	Normal pattern of CS is lost because of atypical sulci pattern and vertical extension of sylvian fissure	IAHR - RH hand motor cortex extends anteriorly into MFG and SFG.	Phase 1 evaluation of refractory Focal epilepsy secondary to cortical brain malformation.	Lacosamide and levetiracetam	non-functional
D6	M	6.0	LH mesiotemporal cortical dysplasia	Intact motor cortex	IAHR - LH hand motor cortex extends anteriorly into MFG and SFG.	Phase I evaluation of intractable, symptomatic localization-related epilepsy secondary to LH lesion	Lacosamide, clobazam, and diazepam	functional
D7	M	7.0	RH polymicrogyria in middle and inferior frontal gyri and portions of temporal lobe	> 50% damage to motor cortex, sparing medial parts of CS	IAHR - RH hand motor cortex Localized within leg motor area	Phase I evaluation of refractory symptomatic partial epilepsy from right hemisphere secondary to polymicrogyria.	Levetiracetam	functional

D8	M	7.3	LH extensive subependymal gray matter heterotopia, periventricular and subcortical nodular heterotopia extending along the frontal lobe and parietal lobe; loss of gray-white matter differentiation in left superior, inferior and middle frontal gyri; thickening and irregularity of the left frontal and parietal lobes including perirolandic cortex with suggestion of polymicrogyria extending posteriorly along the left insula, and perisylvian cortex; white matter volume loss in left cerebral hemisphere.	Abnormal gyration/sulcation pattern in CS	IAHR - LH hand motor cortex Localized anterolaterally within IFG over the area of polymicrogyria	Phase I evaluation of refractory symptomatic partial epilepsy from left hemisphere secondary to polymicrogyria.	Topiramate, zonisamide, lacosamide, and oxcarbazepine	functional
D9	M	7.7	Bilateral perisylvian and parietal polymicrogyria and bilateral occipital subependymal heterotopic gray matter; decreased parietooccipital white matter volume bilaterally.	Loss of normal pattern of CS because of polymicrogyria in both hemispheres; vertical extension of the sylvian fissures bilaterally, which posteriorly appear as deep sulci in the parietal lobes.	IAHR: anterior to putative central sulcus in both hemispheres	Diagnostic evaluation of intractable, symptomatic partial seizures related to structural brain malformation.	Gabapentin	functional (R < L)
D10	M	9.1	Bilateral Polymicrogyria (LH > RH) from the sylvian fissures extending posteriorly and superiorly along the parietal occipital sulci bilaterally; polymicrogyria is in close proximity to the inferior lateral aspects of the perirolandic regions but not extending to the expected anatomic region of the primary sensory and motor regions.	Loss of normal pattern of CS because of polymicrogyria in both hemispheres.	IAHR - hand motor cortex in both hemispheres extends medially into SFG and leg primary motor cortex	Diagnostic evaluation of intractable, symptomatic partial seizures related to structural brain malformation.	Zonisamide, lacosamide, and valproic acid	functional
D11	M	10.0	RH parietooccipital cortical dysplasia s/p right temporal, occipital and posterior parietal lobectomy with gliosis along the anterior margin of the resection cavity within the postcentral gyrus.	Intact motor cortex	IAHR: - hand motor cortex in RH extends posterolaterally and inferiorly over postcentral gyrus	Diagnostic evaluation of intractable focal epilepsy s/p resection involving the right parietal, occipital, and temporal lobes.	None	non-functional
D12	F	11.8	LH temporal porencephalic cyst; asymmetrically decreased volume of cerebral peduncle; cortical organizational abnormality in the posterior frontal lobe; atypical sulcation pattern in the perirolandic region without discrete identification of a typical appearing central sulcus.	Normal pattern of CS is lost because of atypical sulci pattern in LH	IAHR - hand motor cortex in left hemisphere extends medially into SFG and leg primary motor cortex	Phase I evaluation of Intractable, symptomatic partial onset seizures of left hemisphere origin	Lacosamide and lamotrigine	functional

D13	F	12.2	RH s/p frontal topectomy and corpus callosotomy; Chromosomal encephalopathy (microduplication Xp 11.4 – p11.24) with hydrocephalus.	Intact motor cortex	IAHR - hand motor cortex in right hemisphere displaced posteriorly, over postcentral gyrus	Phase I evaluation of intractable, symptomatic partial seizures of right hemispheric origin with rare seizures of left hemisphere origin.	Lacosamide, levetiracetam, and felbamate	non-functional
D14	F	13.8	RH Polymicrogyria; dysplasia of the frontal and parietal lobes and insula with volume loss, polymicrogyria, and heterotopic gray matter along the posterior right lateral ventricle. There is a vertical cleft extending from the posterior aspect of the sylvian fissure and volume loss involving thalamus and lentiform nucleus.	Vertical cleft in the region of central sulcus. Motor cortex otherwise intact	IAHR - hand motor cortex in right hemisphere displaced laterally to face/mouth motor area and anteriorly to MFG	Phase I evaluation of intractable symptomatic epilepsy, focal due to RH malformation.	Oxcarbazepine and clonazepam	functional
D15	F	13.8	Tuberous sclerosis complex with stigmata of tuberous sclerosis complex; LH area of calcification and cortical dysplasia in superior and middle frontal gyri; prior focal resection involving postcentral gyrus, with minimal involvement of the precentral gyrus.	Intact motor cortex	IAHR - LH hand motor cortex extends anteriorly into MFG and SFG.	Phase I evaluation of intractable, symptomatic, complex partial seizures of left hemisphere origin secondary to TSC.	Zonisamide and febamate	non-functional
D16	F	18.1	RH focal bottom of the sulcus cortical dysplasia in paracentral lobule	Intact hand motor cortex, dysplasia in leg primary motor cortex.	IAHR - RH hand motor cortex extends anteriorly into MFG and IFG; leg primary motor cortex displaced to hand motor cortex area	Phase I evaluation of intractable symptomatic seizures secondary to dysplastic cortex in RH.	Lacosamide and clobazam	functional
D17	F	19.1	RH polymicrogyria in anterior middle frontal gyrus; Open with schizencephalic cleft contiguous with the anterior body of the right lateral ventricle. The cleft is lined by gray matter with polymicrogyria.	Middle and lateral parts of precentral gyrus affected by polymicrogyria	IAHR - RH hand motor cortex extends anteriorly into MFG and IFG	Phase I evaluation of intractable symptomatic partial and secondary generalized seizures of right frontal lobe origin	Topiramate, oxcarbazepine, and lorazepam	non-functional
D18	F	7.9	RH polymicrogyria involving the perirolandic region, inferior frontal gyrus, inferior parietal lobule, anterior aspect of superior parietal lobule, superior temporal gyrus and superior margin of the posterior portion of the right middle temporal gyrus. There is a superior extension of the right sylvian fissure.	Intact motor cortex	None: motor cortex normally localized in CS in both hemispheres. Premature pattern of motor map in RH with bilateral representation	Phase I evaluation of intractable symptomatic partial seizures localized to RH posterior-lateral parietal lobe. Secondary to polymicrogyria.	Oxcarbazepine and levetiracetam	non-functional
D19	F	2.1	RH cortical dysplasia in posterior middle and inferior frontal gyri s/p surgical resection	Intact motor cortex	None: motor cortex localized in the central sulcus in expected location in both hemispheres.	Diagnostic evaluation of new paroxysmal events with h/o resolved symptomatic partial seizures of right hemisphere origin following resection of cortical dysplasia	Zonisamide and oxcarbazepine	functional

f - female; m - male; LH - left hemisphere; RH - right hemisphere; TSC: Tuberous sclerosis complex; s/p: status post; SMG: supramarginal gyrus; MFG: middle frontal gyrus; SFG: superior frontal gyrus; IPL: inferior parietal lobule; IFG: inferior frontal gyrus; M1: primary motor cortex; CS: Central sulcus
